# Cyclodextrins-in-Liposomes: A Promising Delivery System for *Lippia sidoides* and *Syzygium aromaticum* Essential Oils

**DOI:** 10.3390/life12010095

**Published:** 2022-01-10

**Authors:** Iara Baldim, Andressa M. Oliveira, Eliana B. Souto, Wanderley P. Oliveira

**Affiliations:** 1 School of Pharmaceutical Sciences of Ribeirão Preto, University of São Paulo, Ribeirão Preto 14040-903, SP, Brazil; iara.baldim@usp.br (I.B.); andressa.matias.oliveira@usp.br (A.M.O.); 2 CEB—Centre of Biological Engineering, University of Minho, Campus de Gualtar, 4710-057 Braga, Portugal

**Keywords:** proliposomes, cyclodextrins, thymol, eugenol, *Lippia sidoides*, *Syzygium aromaticum*

## Abstract

Biological activity of essential oils (EOs) has been extensively reported; however, their low aqueous solubility, high photosensitivity, and volatility compromise a broad industrial use of these compounds. To overcome these limitations, we proposed a nanoencapsulation approach to protect EOs, that aims to increase their stability and modulate their release profile. In this study, drug-in-cyclodextrin-in-liposomes encapsulating two essential oils (*Lippia sidoides* and *Syzygium aromaticum*) and their respective major compounds (thymol and eugenol) were produced by ethanol injection and freeze-dried to form proliposomes and further physicochemically characterized. Liposomes showed high physical stability over one month of storage at 4 °C, with slight changes in the mean size, polydispersity index (PDI), and zeta potential. Reconstituted proliposomes showed a mean size between 350 and 3300 nm, PDI from 0.29 to 0.41, and zeta potential between −22 and −26 mV. Differential scanning calorimetry and X-ray diffraction of proliposomes revealed a less-ordered crystalline structure, leading to high retention of the major bioactive compounds (between 73% and 93% for eugenol, and 74% and 84% for thymol). This work highlights the advantages of using drug-in-cyclodextrin-in-liposomes as delivery systems to retain volatile compounds, increasing their physicochemical stability and their promising potential to be utilized as carriers in products in the pharmaceutical, food, and cosmetic industries.

## 1. Introduction

Essential oils (EOs) are known for showing a multitude of biological effects due to their variable terpene, terpenoid, and phenolic contents [1]. Numerous studies have highlighted the properties of EOs as antimicrobial [2,3,4], antiviral [5], pesticide [6], larvicidal [7], anti-inflammatory [8], and antioxidant [9] agents, among others. These compounds are widely accepted by consumers because of their natural status and safety profile [1]. In particular, the essential oils of *Lippia sidoides* (LEO), popularly known as pepper rosemary, and clove (*Syzygium aromaticum*) (CEO) have been extensively studied for their antimicrobial effects [2,10]. These EOs are rich in thymol and eugenol, respectively, compounds that are linked to their antimicrobial activity. Despite the high potential use as active ingredients, EOs are volatile liquids, very sensitive to environmental variations; and susceptible to degradation, especially when exposed to light, oxygen, heat, and humidity. To widen their applicability, encapsulation technologies have been proposed [2,11,12].

Liposomes have been widely used as drug delivery systems for sustained-release purposes. They are non-toxic lipid-based carriers consisting of concentric vesicles formed by one or more phospholipid bilayers [13,14]. The amphiphilic character of phospholipid molecules offers the liposomes the ability to encapsulate both hydrophilic and lipophilic compounds, being an attractive approach for the loading of EOs [15]. In contrast, problems such as oxidation and hydrolysis of the phospholipids lead to leakage of the encapsulated active ingredient and aggregation of the vesicles [16]. Moreover, the entrapment of lipophilic compounds is limited to the inner hydrocarbon chains of the lipid bilayers, and can thus be rapidly released from the liposomes [17]. Cyclodextrins (CDs), on the other hand, are suitable to form inclusion complexes with a large variety of molecules due to their ability to establish weak intermolecular interactions and increase the solubility of lipophilic compounds [18], both in the solid and aqueous state [19,20]. This process has, as its main advantages, high encapsulation efficiency and long retention time. The possibility of transforming liquid compounds into crystalline forms, masking possible odors and unpleasant tastes, and increasing the physicochemical stability of volatile compounds are other notable advantages [20]. It is also noteworthy that CDs are generally recognized as safe by the United States Food and Drug Administration (US FDA) agency for use as protectants and additives in food products, and also as flavor carriers [20].

Beta-cyclodextrin (β-CD) is a cyclic polysaccharide, which presents a hydrophilic external surface and a hydrophobic internal cavity [6]. Its derivative 2-hydroxypropyl-β- cyclodextrin (HP-β-CD) has attracted interest due to its improved ability to form inclusion complexes, greater water solubility, and lower toxicity toward biological membranes [21]. Furthermore, the presence of CDs in the aqueous core of liposomes preserves the integrity of the membrane without affecting the characteristics of the liposomes. Hence, drug-in-cyclodextrin-in-liposomes (DCL) can be an interesting approach, since it combines the advantages of both carriers, cyclodextrins, and liposomes, in providing controlled drug release [21]. DCLs are based on the entrapment of CD/drug inclusion complex into the inner cavity of liposomes. This technique allows an increase in the solubility of the bioactives and in the stability of the vesicles; being particularly suitable for the delivery of volatile compounds [20], like essential oils. Additionally, compared to conventional liposome production methods, the double loaded technique provides rapid release of the actives from the outer phospholipid bilayer, as well as prolonged release due to the presence of the inclusion complex in the inner aqueous core [22].

To ensure the stability and increase the shelf-life of liposomes containing volatile compounds, freeze-drying can be used very conveniently. Such drying processes occur at low temperatures and without the presence of water in the liquid form, favoring both greater stability and the retention of bioactives. The particulate-based forms, known as proliposomes, generate liposomal suspension upon hydration under appropriate stirring conditions [23,24].

Although many recent studies have used DCL as carriers for encapsulating lipophilic compounds [18,21,25,26], few attempts have been made on the encapsulation of more than one essential oil in the same structure. This is a challenging strategy as EOs have a complex and varied composition, including terpenes, phenylpropenes, and oxygenated compounds. There can be over a hundred different compounds in a single EO [1]. Despite presenting challenges in the production stage, the inclusion of more than one bioactive (or more than one EO) has the possibility of a producing a synergistic effect and can potentially reduce the dose required for single drug usage with increased drug-efficacy, and subsequently lower drug toxicity.

Therefore, the purpose and the novelty of the present study is the development of proliposomes of DCLs combining two EOs (or the combination of their major isolated bioactives). The proliposomes of DCLs were prepared by the double loading technique, where the bioactives were added both in the organic phase and in their inclusion complex in the aqueous phase. We used different bioactives combinations (both *Lippia sidoides* and clove essential oils in combination and isolated, and their respective major components, thymol, and eugenol). Based on its remarkable antimicrobial activity, the pepper rosemary and clove EOs, and their major compounds, thymol, and eugenol, were chosen as models for this study. Proliposomes were obtained by freeze-drying, and their reconstituted liposomes were characterized concerning the mean hydrodynamic diameter, polydispersity index, zeta potential, differential scanning calorimetry, X-ray diffraction, and retention of the bioactive major compounds.

## 2. Materials and Methods

### 2.1. Materials

2-Hydroxypropyl-β-cyclodextrin (HP-β-CD) was supplied by Roquette (Lestrem, France), and hydrogenated soybean Phospholipon 90H was purchased from Lipoid GmbH (Ludwigshafen am Rhein, Germany). Eugenol (Eug), eugenyl acetate (Eug-Ac), thymol, absolute ethanol, cholesterol, and methanol-HPLC grade were purchased from Sigma–Aldrich (Darmstadt, Germany). CEO (essential oil from *Syzygium aromaticum*), having as the main compounds Eug (86.89%), Eu-Ac (2.91%), trans-caryophyllene (9.04%), α-caryophyllene (0.97%), and the butyl acetate (0.09 %), was bought from a clove essential oil producer located in Valença (BA, Brazil). LEO (essential oil from *Lippia sidoides*), having thymol (68.5%), p-cimeno (9.43%), trans-caryophyllene (7.72%), β-myrcene (2.84%), γ-terpinene (2.71%), α-terpinene (1.16%), and thymol methyl ether (0.97%) as the main compounds, was obtained from PRONAT (Produtos Naturais Ltd.a, Recife, PE, Brazil).

### 2.2. Preparation of Drug-in-CD-in-Liposomes

Liposomal formulations were prepared by the ethanol injection method adapted from Sebaaly et al. [17]. Briefly, the aqueous phase, consisting of the inclusion complexes of HP-β-CD with bioactives was prepared by dissolving HP-β-CD in ultra-pure water and adding the bioactive (CEO and/or LEO, eugenol, and thymol). This solution was stirred for 24 h at 25 ± 2 °C. Subsequently, the organic phase was prepared by dissolving Phospholipon 90H and cholesterol in absolute ethanol under magnetic stirring and heated at 55 °C (above the transition temperature of the phospholipids), when the previously heated bioactive (CEO, LEO, thymol, and Eug) was added. A peristaltic pump was used to inject the organic phase into the aqueous phase at a flow rate of 1 mL/min. The obtained dispersion was kept under magnetic stirring for 15 min, at room temperature. Finally, ethanol and some of the water were removed by rotary evaporation at 45 °C (Rotavapor Fisatom model 802, Perdizes, SP, Brazil). The storage of all dispersions prepared took place in a dark room at 4 °C. Table 1 shows the composition of the produced EOs-in-cyclodextrin-in-liposomes.

### 2.3. Preparation of Proliposomes

**Figure 1:**
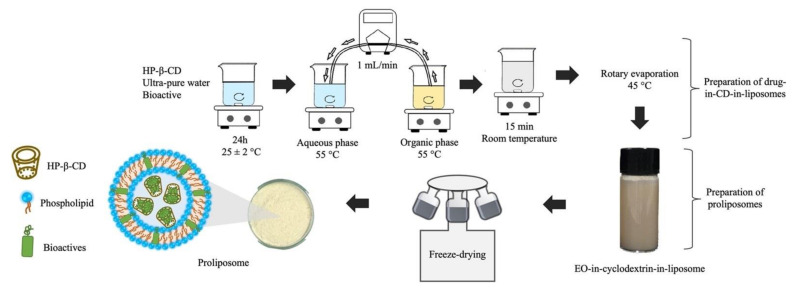
Diagram of EOs-in-cyclodextrin-in-liposome and proliposomes production.

Freshly prepared liposomal suspensions were frozen at −20 °C for about 12 h in plastic tubes (50 mL) and for a further 4 h at −80 °C before freeze-drying (MicroModulyo, Thermo Fisher Scientific, Waltham, MA, USA). Lyophilization time lasted for 72 h. The lyophilized liposomes were stored at −20 °C for further analysis and characterization. Figure 1 shows the production scheme of EO loaded carriers.

### 2.4. Mean Hydrodynamic Diameter, Polydispersity Index, and Zeta Potential

The mean hydrodynamic diameter and polydispersity index of the EOs-in-cyclodextrin-in-liposomes were determined by dynamic light scattering (DLS) using a Zetasizer Nano ZS90 (Malvern, UK). Zeta potential was measured by micro electrophoresis, with the same equipment. All the liquid samples were diluted in MilliQ^®^ water (Millipore, Billerica, MA, USA) at a ratio of 1:10 (v/v) and measured in triplicate at 25 °C. The measurements were repeated on the 1st, 15th, and 30th days after production. For the analysis of proliposomes, these were firstly redispersed in MilliQ^®^ water at the original concentration, stirred for 30 min, and diluted to 1:200 (v/v) before measurements.

### 2.5. Differential Scanning Calorimetry

A differential scanning calorimeter (Shimadzu DSC-50, Shimadzu Corporation, Kyoto, Japan) was used to study the thermal transformations of the components of the formulations and their relationship with particle structure, according to the method described by Zhang et al., with some modifications [27]. Measurements of each component of the formulations, of the physical mixture, and the proliposomes were performed. The samples were exactly weighted in aluminum pans and heated from 20 °C to 250 °C following cooling to 20 °C at a heating and cooling rate of 10 °C/min.

### 2.6. X-ray Diffraction Study

The X-ray powder diffraction patterns were obtained using a Rigaku Rotaflex RU200B X-ray diffractometer (Tokyo, Japan) with Cu-Kα radiation (λ = 1.5418 Å). Samples were scanned at a current of 100 mA and a voltage of 50 kV. Patterns were obtained using a step width of 1.2°/min from 0° to 50° at room temperature on a 2θ scale.

### 2.7. Retention of Bioactives

The amount of the major bioactive compounds (eugenol and thymol) in the liquid and dried products was monitored by high-performance liquid chromatography with diode array detection (HPLC-DAD), following a method previously developed by Leal et al. [28] and validated by our group. Analyses were performed in a Shimadzu HPLC (Shimadzu Corporation, Kyoto, Japan) using a C-18 column (Shimadzu Shim-Pack CLC(M) 4.6 mm × 25 cm, 5 μm, 100 Å) at an oven temperature of 30 °C, with a volume injection of 20 µL. The mobile phase used was water (A) and acetonitrile (B) using gradient elution: 0–2 min 10% B in A; 2–7 min 10–78% B in A; 7–17 min 78% B in A; 17–20 min 78–100% B in A; 20–23 min 100% B in A; 23–26 min 100–10% B in A; and 23–32 min 10% B in A. The chromatograms were recorded at 276 nm. The samples were diluted with methanol, homogenized in an ultrasound bath, and kept under magnetic stirring for 30 min. After the extraction, the samples were centrifuged for 5 min at 5000× *g* and the supernatants were filtered in a 0.4 µm Millipore membrane and analyzed by HPLC.

### 2.8. Statistical Analysis

Results were expressed as mean ± SD. A two-way analysis of variance (ANOVA) using a Bonferroni post-hoc test was used to compare the levels of significance between the samples and *p* < 0.05 was considered statistically significant.

## 3. Results and Discussion

### 3.1. Stability Analysis of Liposomes

**Figure 2:**
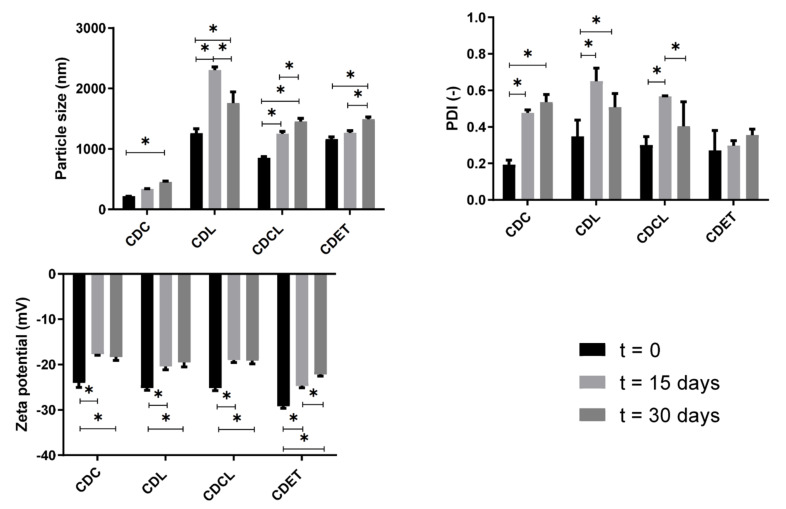
Stability parameters of EOs-in-cyclodextrin-in-liposome systems during storage at 4 °C, at t = 0 days, t = 15 days, and t = 30 days. The symbol (*) means significant difference at *p* < 0.05 (Bonferroni post-hoc test).

For most commercial applications the long-term stability is an important parameter to be considered for a delivery system. We, therefore, carried out a series of tests to evaluate the physicochemical stability of liposomes over one month. The mean hydrodynamic diameter, PDI, and ZP of the liposomal formulations were measured over a storage period of 30 days at 4 °C. The freshly prepared liposomal solutions exhibited a homogeneous whitish appearance. Figure 2 demonstrates the distribution of nanometric and micrometric particles, varying according to the encapsulated bioactive. In general, the systems showed an appreciable increase in the hydrodynamic diameter throughout 30 days of storage at 4 °C, which suggests vesicle coalescence. On the other hand, the liposome formulation containing clove essential oil (CDC) had a mean particle size perceptibly smaller than the other samples. We hypothesized that the differences in particle size are at least partly due to the type of the encapsulated essential oil, because we obtained larger liposomes containing *Lippia sidoides* essential oil when compared to the liposomes containing clove essential oil, produced by the same method in our pre-formulation studies (data not shown).

The polydispersity index (PDI) was determined to indicate the width of particle size distribution, whose values ranges from 0 (monodisperse system) to 1 (very polydisperse distribution), reflecting the tendency of the particles to aggregate. The PDI values were similar for all the formulations, slightly increasing upon storage. Similar findings were obtained by other authors [29,30], possibly because HP-β-CD might replace the drug molecules from the hydrophobic core with cholesterol (or another lipid component), favoring the destabilization of the liposomal structure [31]. This problem is based on a question of affinity of the asset with the HP-β-CD, the greater the affinity of the bioactive for the HP-β-CD, the more stable the inclusion complex will remain. Therefore, to overcome this problem, the types and concentrations of CD, and the affinity with the drug molecules should be intensively investigated during the pre-formulation studies. The selection of lipids with lower affinity for cyclodextrin than cholesterol or the drug itself should be preferable [31]. As an indicator of vesicle stability, ZP values were reduced over the storage time, corroborating the evidenced changes in particle size and PDI.

### 3.2. Proliposomes Properties

Reconstituted proliposomes were characterized and compared in terms of mean diameter, PDI, and ZP (Table 2). Significant differences were found between the sizes of the liposomal systems. The presence of LEO affected the reconstituted liposomes mean size significantly; CDL and CDET showed similar sizes. PDI was also similar among the developed formulations, slowly increasing from 0.3 to ≈ 0.4, indicating the relative homogeneity of proliposomes. The ZP is a parameter linked to the electrostatic repulsion between suspended or emulsified systems and generally is used as indicative of system stability [32]. All formulations showed negative values of zeta potential, ranging from −22.3 to −26.5 mV. Particularly, the larger proliposomes (CDCL) showed significantly higher zeta potential than the others, in agreement with literature results [32].

Determination of an inclusion complex between host and guest molecule depends on a variety of parameters and requires multivariate analysis for this description. Therefore, complementary characterization methods, including differential scanning calorimetry (DSC) and X-ray diffraction (XRD), were employed to verify the occurrence of complexation. DSC is the most commonly used technique for determining the thermal effects of the material. It quickly provides accurate information on both the physical and energetic properties of the material [33] and indirect evidence about the formation of the cyclodextrin inclusion complex [6]. In this study, DSC was used to study the influence of bioactive compounds on lipid membrane organization. 

**Figure 3:**
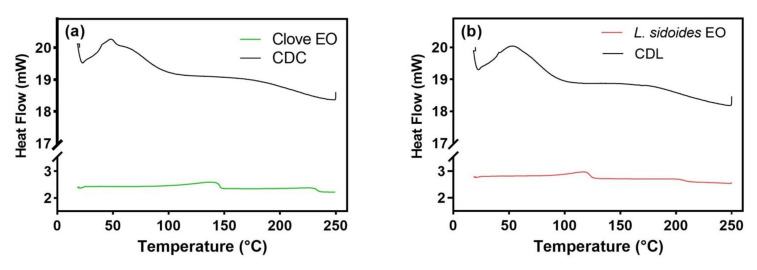

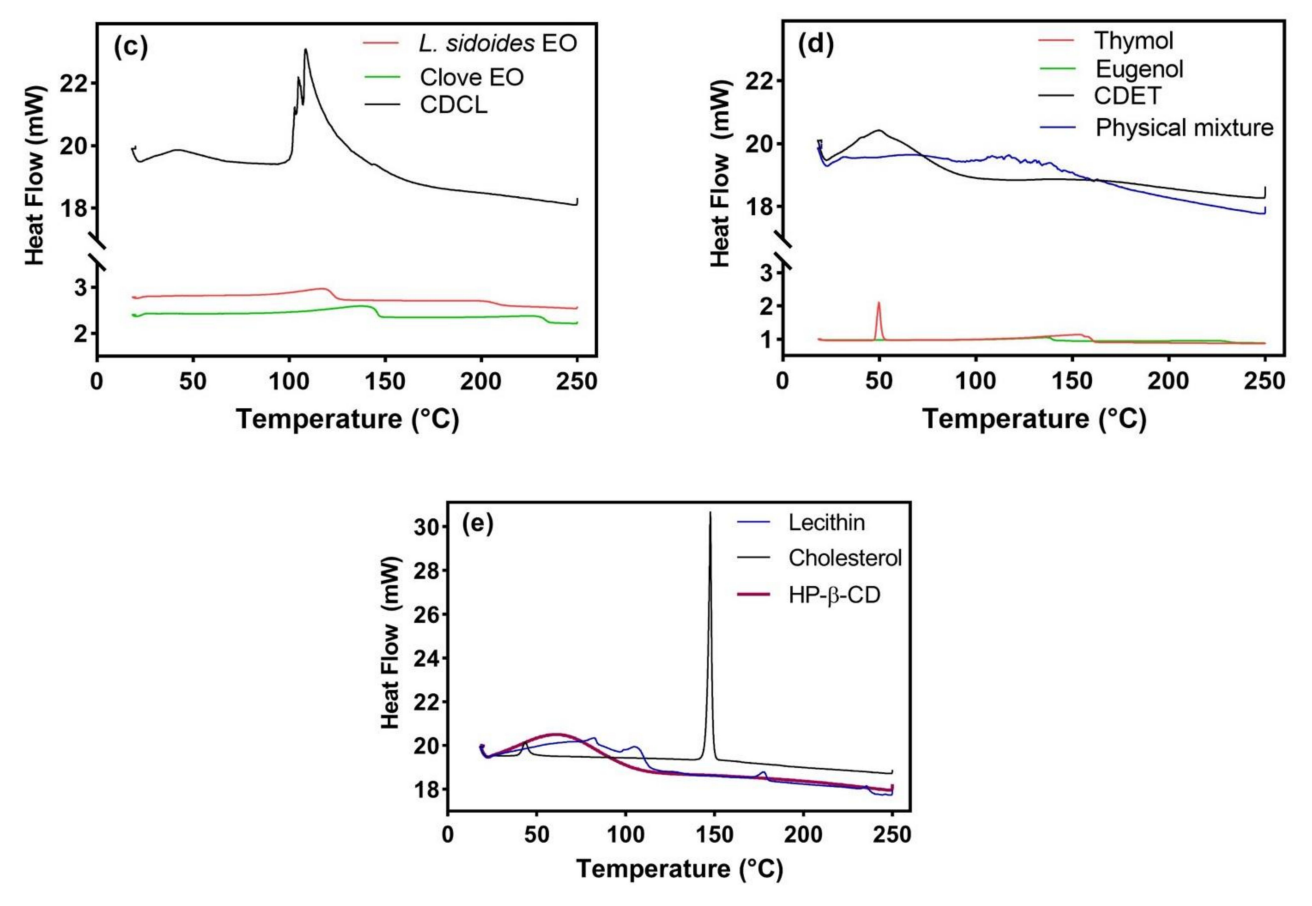
DSC thermograms of proliposomes, essential oils, isolated bioactives, and raw materials: (**a**) CDC proliposome loaded by clove essential oil and pure clove essential oil; (**b**) CDL proliposome loaded by *L. sidoides* essential oil and pure *L. sidoides* essential oil; (**c**) CDCL proliposome loaded by both essential oils (*L. sidoides* and clove), and the pure essential oils; (**d**) CDET proliposome loaded by both isolated bioactives (thymol and eugenol), the pure isolated bioactives, and physical mixture; (**e**) bulk raw materials (lecithin, cholesterol, and HP-β-CD). Up: endothermic heat flow.

It is well known that some constituents of the essential oils, among them thymol and eugenol, can increase the fluidity of the liposomal membrane by reducing the phase transition temperature of the phospholipid [34,35]. The DSC analysis of the proliposomes (Figure 3 and Table 3) showed no melting peak of the actives (represented by red or green lines in Figure 3), indicating the absence of any significant level of crystallinity in the analyzed product, confirming its amorphous state. Moreover, the bioactive compounds were able to interact with the phospholipidic membrane, causing variations in the thermodynamic parameters (peak temperature—T_m_ and enthalpy difference—∆H) (Table 3). Such variations were more pronounced in proliposomes loading thymol, eugenol, and clove EO. According to Cristani et al. [36], these terpenes act as substitutional impurities, causing a decrease in T_m_ and ∆H values. The disappearance or even gradual decrease of the melting point of a crystalline guest compound provides indirect evidence of the inclusion complex formation with cyclodextrin. However, DSC alone is not a suitable technique to confirm the formation of the cyclodextrin inclusion complex when using volatile substances as guest compounds [37], as the essential oils, thymol, and eugenol. For such guest molecules, X-ray powder diffraction is the most useful technique to detect the inclusion complex formation [20].

**Figure 4:**
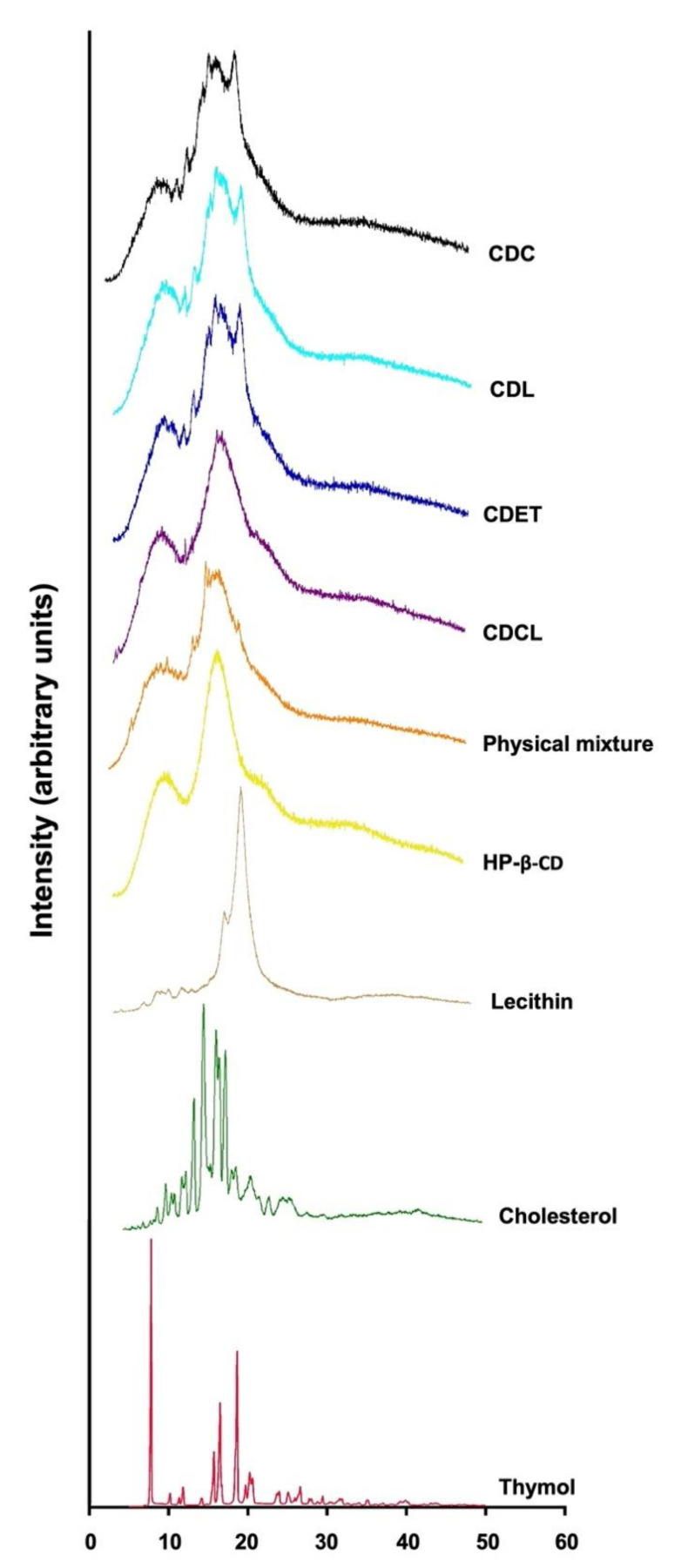
X-ray diffraction (XRD) profiles of proliposomes (CDL, CDCL, CDET), physical mixture (lecithin, cholesterol, HP-β-CD, thymol, and eugenol), and bulk raw components.

X-ray diffraction is also able to characterize and identify the structure of lipid and drug molecules [38]. The X-ray powder diffraction patterns of pure compounds and loaded proliposomes are presented in Figure 4. The peaks patterns from HP-β-CD are also present in the samples (CDC, CDL, CDCL, and CDET) and the physical mixture, but are broader and with lower intensity. The addition of guest compounds to the HP-β-CD increased the lattice disturbance of the latter, as can be seen by comparing the patterns of the HP-β-CD with the proliposomes. This interaction resulted in the appearance of new peaks in the proliposome samples, producing a diffractogram that differs from the one of non-complexed HP-β-CD, evidencing the formation of the inclusion complexes. Concerning lecithin, its main peak was presented in CDC, CDL, and CDET proliposomes, also with a decrease in intensity. The decrease in the intensity of this peak may indicate a change in the crystalline structure of lecithin and a less ordered structure of proliposomes. The broad diffraction peaks also reflect a reduction in crystallinity [6,39]. Such an amorphous state might contribute to the higher retention of bioactives [39]. However, as the diffraction patterns presented by CDC, CDL, and CDET proliposomes were similar, retention of bioactives in these samples probably also remained similar.

### 3.3. Retention of Marker Compounds in Proliposomes

**Figure 5:**
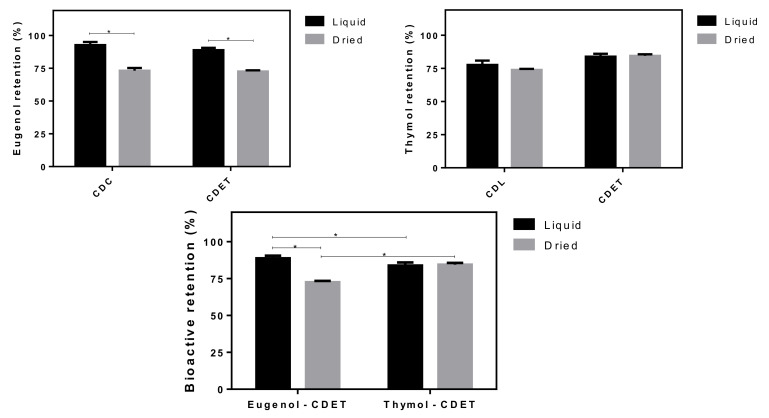
Eugenol and/or thymol retention of liquid and freeze-dried forms of EOs-in-cyclodextrin-in-liposome systems. The symbol (*) means significant difference at *p* < 0.05 (Bonferroni post-hoc test).

The content of bioactives in the proliposomes is strongly associated to the previously investigated variables. The results of eugenol and thymol retention in the liposomal structures are shown in Figure 5. The eugenol retention reached near 90% for liquid samples and 72% for proliposomes, while for thymol the values were around 80% for both systems, with no statistical difference between the samples (*p* < 0.05). Indeed, the freeze-drying had a significant influence on eugenol retention both for formulations containing essential oil (CDC and CDL) and the isolated bioactives (CDET). Furthermore, the liquid formulations achieved higher eugenol retention in comparison to thymol, but the effect was significantly lower for the dried proliposomes. Similar results for eugenol loss during the freeze-drying of β-CD inclusion complexes have also been reported [6]. The relatively higher lipophilicity of thymol suggests favored partitioning both into the lipidic wall of proliposome and the hydrophobic internal cavity of HP-β-CD, rather than the external hydrophilic surface of the cyclodextrin. Hence, thymol is apparently better incorporated into the lipophilic parts of the delivery system, remaining retained, despite the water removal during the freeze-drying process. Molecular modelling studies could explain the differences between eugenol retention in liquid and proliposomes samples through the arrangement of bioactive molecules inside the CD cavity. On one hand, the eugenol molecule enters into the CD cavity from the wider rim, leaving OH and MeO groups outside and the allyl group keeps folded towards the phenyl ring [40]. On the other hand, the thymol molecule is located inside the hydrophobic cavity of CD in a way that allows its OH group to come close to the hydroxyl groups of CD to form hydrogen bonds that further stabilize the inclusion complex [41]. Thus, thymol tends to be more stabilized in the inclusion complex when compared to eugenol, despite the negative pressure and water removal from the freeze-drying drying process.

## 4. Conclusions

Taken together, the results described in the present study allow us to potentially propose drug-in-cyclodextrins-in-liposome as a carrier system to be loaded with *Lippia sidoides* and/or *Syzygium aromaticum* EOs, as well as the isolated EOs main compounds eugenol and thymol. With the selected preparation method relatively stable particles and proliposomes have been obtained. The liquid systems and reconstituted composition showed sizes between 350 nm and 3300 nm with a low polydispersity index and zeta potential around −23 mV. The samples also presented a low degree of crystallinity and high retention of the major marker compounds (eugenol and thymol). While not compromising the objectives of this study, which have been successfully accomplished, namely the demonstration that our proposed methodology has been successful for the encapsulation of EOs and other natural phytopharmaceutical and volatile compounds in cyclodextrin-in-liposomes, we share the opinion that some additional studies, namely, the assessment of the release and toxicological profiles, including scanning/transmission electron microscopy and Fourier-transformed infrared spectroscopy, as well as the evaluation of the promising biological activity, will certainly add value to the potential use of these systems. Overall, EOs-in-cyclodextrin-in-liposome has remarkable potential for use in a wide variety of products of food, cosmetic, and pharmaceutical industries.

## Figures and Tables

**Table 1:** Composition of the EOs-in-cyclodextrin-in-liposome systems.

Phase	Component (g)	Formulation			
		CDC	CDL	CDCL	CDET
Organic phase	Phospholipon 90H	0.4	0.4	0.4	0.4
	Cholesterol	0.2	0.2	0.2	0.2
	*L. sidoides* EO	-	0.1	0.05	-
	Clove EO	0.1	-	0.05	-
	Thymol	-	-	-	0.05
	Eugenol	-	-	-	0.05
	Ethanol	31.6	31.6	31.6	31.6
Aqueous phase	HP-β-Cyclodextrin	2	2	2	2
	*L. sidoides* EO	-	0.33	0.165	-
	Clove EO	0.27	-	0.135	-
	Thymol	-	-	-	0.11
	Eugenol	-	-	-	0.12
	Water	80.0	80.0	80.0	80.0

**Table 2:** Particle size, PDI, and zeta potential of proliposomes obtained by freeze-drying.

Formulation	Particle Size (nm)	PDI (-)	Zeta Potential (mV)
CDC	354.3 ± 27.1 ^a^	0.41 ± 0.05 ^†^	−22.3 ± 0.7 *
CDL	1648.0 ± 106.4 ^b^	0.41 ± 0.05 ^†^	−23.3 ± 0.6 *
CDCL	3300.3 ± 476.8 ^c^	0.31 ± 0.21 ^†^	−26.5 ± 1.3 **
CDET	1493.0 ± 70.6 ^b^	0.29 ± 0.02 ^†^	−23.6 ± 0.3 *

Same letter (a, b, c) or symbols (*, **, ^†^) means no significant difference according to Bonferroni’s multiple comparison test (*p* < 0.05).

**Table 3:** DSC parameters of proliposomes, bulk materials, bioactive compounds and essential oils.

Sample	Onset (°C)	Peak (°C)	∆*H* (J/g)	Area (mJ)
CDC	32.1	48.7	80.8	177.8
CDL	31.7	54.3	93.9	197.3
CDCL	107.1	108.6	101.5	314.8
CDET	28.0	49.9	113.2	237.7
Lecithin	97.8	106.5	12.1	31.4
Cholesterol	145.8	147.7	67.7	148.9
HP-β-CD	23.2	63.3	160.3	368.7
Eugenol	110.9	136.6	111.7	234.6
Thymol	47.8	49.8	125.6	314.0
*L. sidoides* EO	91.5	117.7	97.8	195.5
Clove EO	101.5	138.4	156.3	343.9

## Data Availability

Data are available from corresponding authors upon request.
